# ﻿*Leontopodiumnyingchiense* (Asteraceae), a new species from Xizang (Tibet), China

**DOI:** 10.3897/phytokeys.249.136846

**Published:** 2024-11-15

**Authors:** Wen-Qi He, Fang-Yu Zhao, Zhao-Fu Chu, Guo-Zhu Chai, Kai-Hui Zhao, Jing-Qin Tian, Bao-Xin Zhang, Fang-Yuan Zhang, Zhi-Hua Liao, Wei-Lie Zheng, Xiao-Zhong Lan

**Affiliations:** 1 The Provincial and Ministerial Co-founded Collaborative Innovation Center for R & D in Xizang Characteristic Agricultural and Animal Husbandry Resources, The Center for Xizang Chinese (Tibetan) Medicine Resources, Joint Laboratory for Tibetan Materia Medica Resources Scientific Protection and Utilization Research of Tibetan Medical Research Center of Xizang, Key Laboratory of Tibetan Medicine Resources Conservation and Utilization of Tibet Autonomous Region, Xizang Agriculture and Animal Husbandry University, Nyingchi, Xizang 860000, China Xizang Agriculture and Animal Husbandry University Nyingchi China; 2 Tiantong National Forest Ecosystem Observation and Research Station, Shanghai Key Lab for Urban Ecological Processes and Eco-Restoration, School of Ecological and Environmental Sciences, East China Normal University, Shanghai 200241, China East China Normal University Shanghai China; 3 School of Life Sciences, Integrative Science Center of Germplasm Creation in Western China (CHONGQING) Science City and Southwest University, The Provincial and Ministerial Co-Founded Collaborative Innovation Center for R&D in Xizang Characteristic Agricultural and Animal Husbandry Resources, TAAHC-SWU Medicinal Plant Joint R&D Center, Southwest University, Chongqing 400715, China Southwest University Chongqing China

**Keywords:** *Leontopodium*, morphological analysis, new species, Nyingchi, phylogenetic analysis

## Abstract

*Leontopodiumnyingchiense*, a new species of Asteraceae from the Xizang (Tibet) Autonomous Region of China, is described and illustrated here. Morphologically, it is most similar to *L.lingianum* but can be distinguished by the combination of monoecious and dioecious individuals, involucral bracts arranged in 3–5 series (with outer series herbaceous and middle to inner series membranous), fimbriate apices on female florets, rough-edged lobes on male florets, and achenes lacking costae. Phylogenetic analyses further support the separation of this new species from related taxa. Finally, we characterize this new species through both morphological comparisons and molecular analyses.

## ﻿Introduction

*Leontopodium* R.Br. ex Cass. belongs to the Asteraceae, Gnaphalieae, Filagininae (Smissen et al. 2020). It comprises perennial herbs or subshrubs with approximately 60 species worldwide, primarily distributed in mountainous regions of Asia and Eurasia. In China, there are 37 species and 2 varieties of *Leontopodium*, with 16 species being endemic to China (Chen and Bayer 2011). This genus is a common component of alpine meadows and slopes in the western and southwestern regions of China, with the Tibetan Plateau being the center of its species diversity (Safer et al. 2011; Lee et al. 2016; Zhao 2021) and 21 species distributed in Xizang (Chen et al. 2023).

During the fourth national survey of traditional Chinese medicine resources conducted from 2013 to 2015, an unidentified species of Asteraceae was discovered in Lulang Town, Nyingchi City, Xizang (Tibet) Autonomous Region. In August 2024, this species was re-investigated, and additional specimens and molecular materials from leaves were collected. This species is morphologically very similar to *L.lingianum* (Y.L.Chen) Dickoré, but it differs from the latter in several characters. Its involucral bracts are arranged in 3–5 series, with the outer series being herbaceous and the inner and middle series having a membranous texture. The species can be either monoecious or dioecious. The achenes are without costa, puberulous, and papillose at the apex. The female floret corolla has fimbriate apices, and the male floret corolla has lobes that are rough and granulate at the edges. Based on morphological comparisons and phylogenetic analyses, we have identified this taxon as a new species.

## ﻿Materials and methods

### ﻿Morphological assessment

Three sets of specimens (a total of 12 specimens), collected from different individuals in 2015 and 2024 in Lulang Town, Nyingchi City, Xizang (Tibet) Autonomous Region, were examined. Based on a thorough review of the literature and detailed comparisons of specimens, we carefully analyzed the morphological differences in the involucre, florets, and achenes between the new species and *Leontopodiumlingianum*.

### ﻿Phylogenetic analysis

Based on the literature (Blöch et al. 2010; Xu et al. 2023), 24 ETS sequences were downloaded from GenBank for phylogenetic analysis, including 37 from *Leontopodium* and 3 from *Gamochaeta* as an outgroup (Table 1). DNA extraction from the leaf tissue of the new species was performed using the CTAB method, and ETS primers were designed using SnapGene v6.02 (Table 2). PCR amplification was then carried out, and the amplified products were detected by agarose gel electrophoresis. Following detection, the PCR products were submitted to Sangon Biotech (Shanghai) Co., Ltd. (https://store.sangon.com/) for sequencing. All sequences were aligned using MUSCLE v3.8.31 (Edgar 2004) and manually adjusted with PhyDE-1 v0.9971 (Müller 2005). The best nucleotide substitution model was determined using Modeltest v3.7 (Posada and Crandall 1998) based on the Akaike Information Criterion (AIC), with the HKY model selected as the best fit. Maximum likelihood (ML) analyses were inferred with RAxML 7.2.6 (Stamatakis 2006). A Bayesian phylogenetic tree was constructed using MrBayes v3.2.7 (Huelsenbeck and Ronquist 2001). The Bayesian analysis started from a random tree and ran four Markov chain Monte Carlo (MCMC) algorithms, sampling every 100 generations for a total of 1,000,000 generations. The tree visualization was performed using Figtree v1.4.4 (Rambaut 2008).

**Table 1.** GenBank accession numbers and vouchers for the samples used in this study.

Species	Voucher	GenBank accession
*Leontopodiumalpinum* Colmeiro ex Willk. & Lange	Wiedermann, 9282 WU	FJ639981
*Leontopodiumandersonii* C. B. Clarke	Dickoré, 14068 WU	FJ640006
*Leontopodiumartemisiifolium* Beauverd	Dickoré, 14574 WU	FJ640009
*Leontopodiumcaespitosum* Beauverd	Dickoré, 14040 WU	FJ640010
*Leontopodiumcalocephalum* Beauverd	Dobner & Xiao, D01-2395 WU	FJ640012
*Leontopodiumcampestre* (Ledeb.) Hand.-Mazz.	–	OP946418
*Leontopodiumconglobatum* (Turcz.) Hand.-Mazz.	–	OP946419
*Leontopodiumdedekensii* Beauverd	Dobner & al., MD01-2358 WU	FJ640001
*Leontopodiumdelavayanum* Hand.-Mazz.	–	OP946421
*Leontopodiumdiscolor* Beauverd	CCCP (US)	KT865349
*Leontopodiumfangingense* Y.Ling	–	OP946422
*Leontopodiumforrestianum* Hand.-Mazz. ex W.W.Sm. & al.	–	OP946423
*Leontopodiumfranchetii* Beauverd	Dickoré, 14499 WU	FJ639983
*Leontopodiumgiraldii* Diels	–	OP946426
*Leontopodiumhaastioides* Hand.-Mazz.	Dickoré, 9892 WU	FJ640013
*Leontopodiumhimalayanum* DC.	Dickoré, 5228 WU	FJ640004
*Leontopodiumjacotianum* Beauverd	Dobner & al., Md01-2445 WU	FJ639995
*Leontopodiumjaponicum* Miq.	Togasi, 1228W	FJ639984
Leontopodiumjaponicumvar.saxatile Y.S.Chen	–	OP946432
*Leontopodiumjunpeianum* Kitam.	–	OP946435
*Leontopodiumleontopodioides* (Willd.) Beauverd	Narantuja, S-070800 BM	FJ640014
*Leontopodiumlingianum* (Y.L.Chen) Dickoré	Dickoré, 10836 WU	FJ639988
*Leontopodiummicrophyllum* Hayata	Hörandl, 9549 WU	FJ640015
*Leontopodiummuscoides* Hand.-Mazz.	–	OP946437
*Leontopodiumnanum* (Hook.f. & Thomson ex C.B.Clarke) Hand.-Mazz.	Dickoré, 9539 WU	FJ640005
*Leontopodiumnivale* (Ten.) A.Huet ex Hand.-Mazz.	Schneeweiss & Schönswetter, 8926 WU	FJ640017
*Leontopodiumniveum* Hand.-Mazz.	Nie 1087 (KUN)	KT865359
*Leontopodiumochroleucum* Beauverd	Klimes, 03-21-30 WU	FJ639997
*Leontopodiumpusillum* (Beauverd) Hand.-Mazz.	Miehe & al., 98-35212 B	FJ640018
*Leontopodiumsinense* Hemsl.	Dobner & al., MD01-2397 WU	FJ639999
*Leontopodiumsmithianum* Hand.-Mazz.	–	OP946443
*Leontopodiumsouliei* Beauverd	Dobner & al., MD01-2404 WU	FJ639998
*Leontopodiumstoechas* Hand.-Mazz.	Nie 2443 (KUN)	KT865363
*Leontopodiumstracheyi* C.B.Clarke ex Hemsl.	Miehe & Miehe, 98-09509 WU	FJ640020
Leontopodiumcf.stracheyi C.B.Clarke ex Hemsl.	Dickoré, 10529 WU	FJ640022
*Leontopodiumsubulatum* (Franch.) Beauverd	Nie 1074 (KUN)	KT865365
*Leontopodiumwilsonii* Beauverd	–	OP946446
*Gamochaetanorvegica* (Gunnerus) Y.S.Chen & R.J.Bayer	–	OP946406
*Gamochaetapensylvanica* (Willd.) Cabrera	–	OP946407
*Gamochaetasylvatica* (L.) Fourr.	–	OP946409

**Table 2.** ETS primer sequences and PCR reaction conditions.

DNA fragment	Primer	Primer sequences	PCR reaction conditions
ETS	ETS-F	GCGCAACAACTTCCACC	94 °C 5 min; 94 °C 1 min, 54 °C 45s, 72 °C 1 min, 30 cycles; 72 °C 5 min
	ETS-R	GGCAGGATCAACCAGGT	

## ﻿Results

### ﻿Taxonomic treatment

#### ﻿ Leontopodium nyingchiense

Taxon classificationPlantaeAsteralesAsteraceae

X.Z.Lan, W.L.Zheng & W.Q.He sp. nov.

F1EA2E70-C5BE-5490-A011-E86C2A77F97A

urn:lsid:ipni.org:names:77351835-1

##### Type.

**Figure 1.**
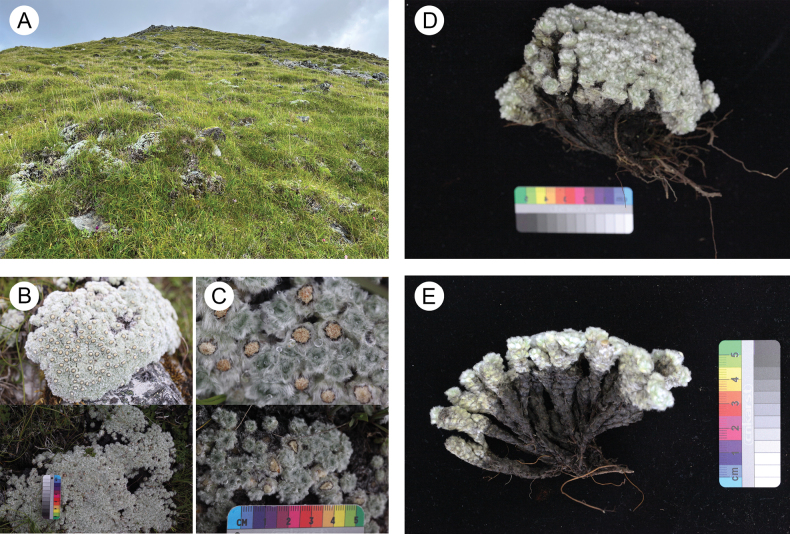
*Leontopodiumnyingchiense* X.Z.Lan, W.L.Zheng & W.Q.He **A** habitat **B, C** morphology in the field **D, E** whole plant.

**Figure 2.**
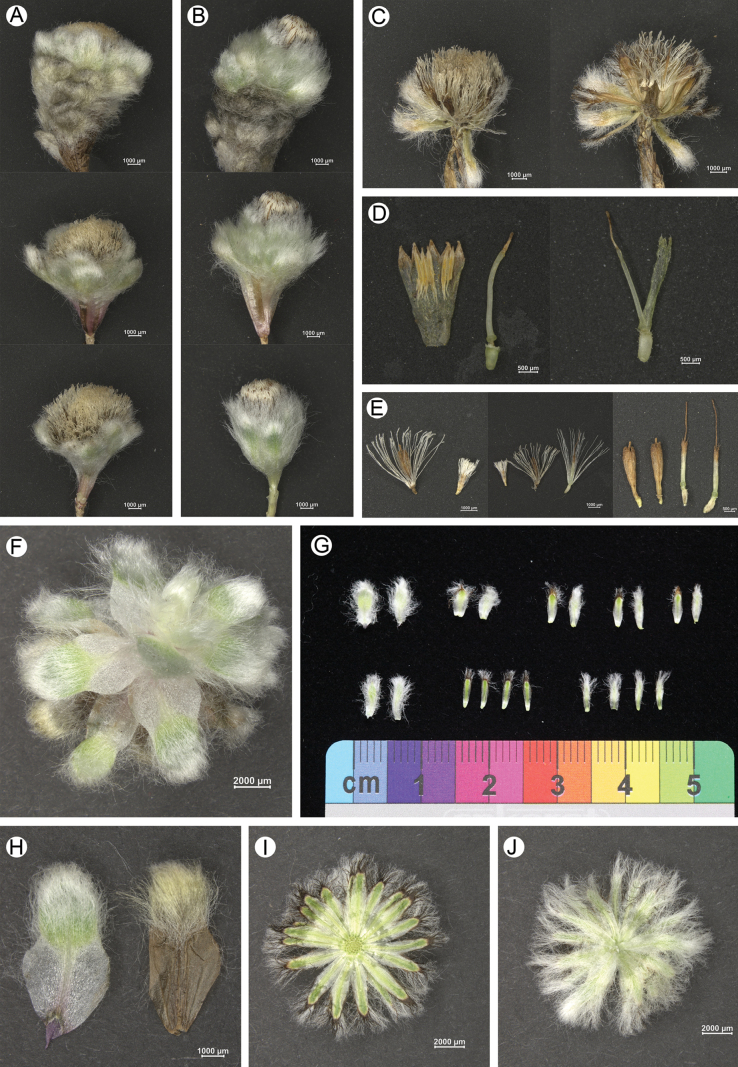
*Leontopodiumnyingchiense* X.Z.Lan, W.L.Zheng & W.Q.He **A** male inflorescence **B** female inflorescence **C** dissected male inflorescence with female florets **D** dissected floret **E** hermaphroditic floret **F** dissected sterile branch **G** outer and inner involucral bracts **H** leaf **I–J** involucre.

**Figure 3.**
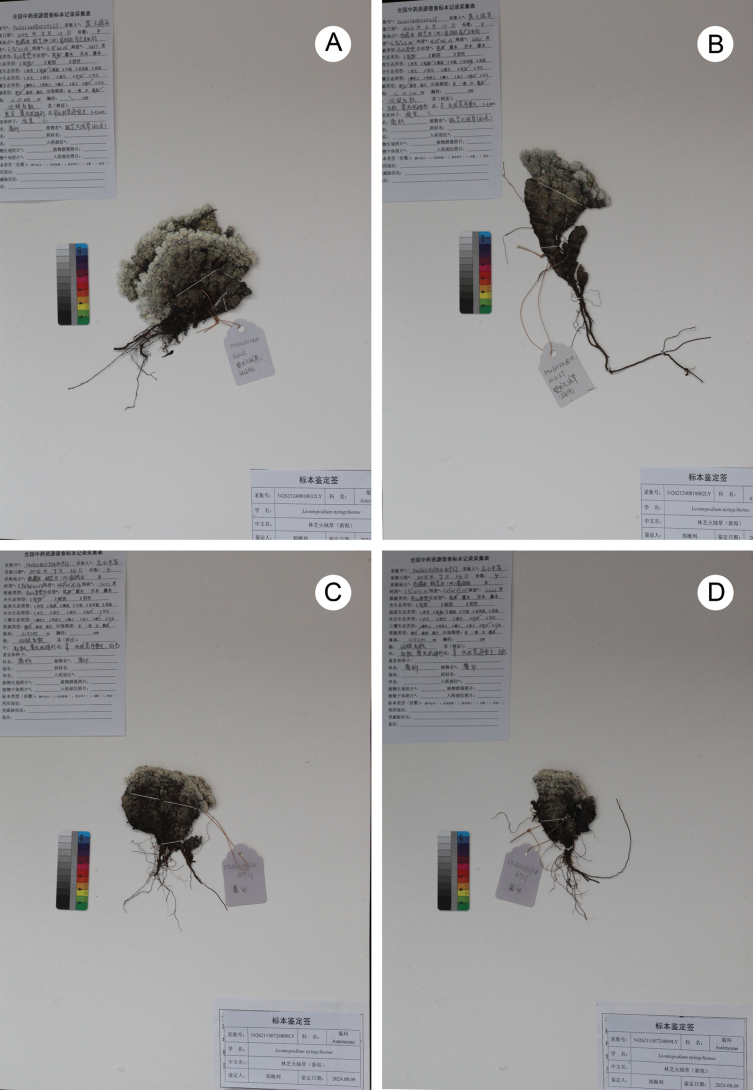
*Leontopodiumnyingchiense* X.Z.Lan, W.L.Zheng & W.Q.He **A** female herbarium specimen **B** male herbarium specimen **C, D** monoecious herbarium specimens (holotype and isotype).

**Figure 4.**
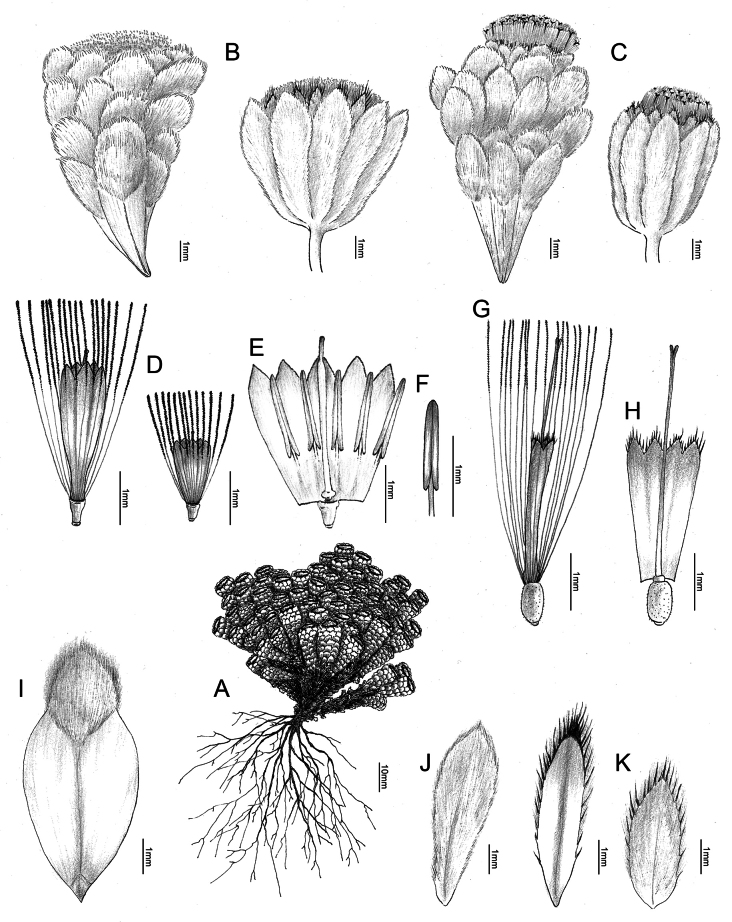
*Leontopodiumnyingchiense* X.Z.Lan, W.L.Zheng & W.Q.He **A** whole plant **B** male inflorescence **C** female inflorescence **D** male floret **E** dissected male floret **F** stamen **G** female floret **H** dissected female floret **I** leaf **J** outer involucral bract **K** middle and inner involucral bracts (drawing by Wenqi He).

**Figure 5.**
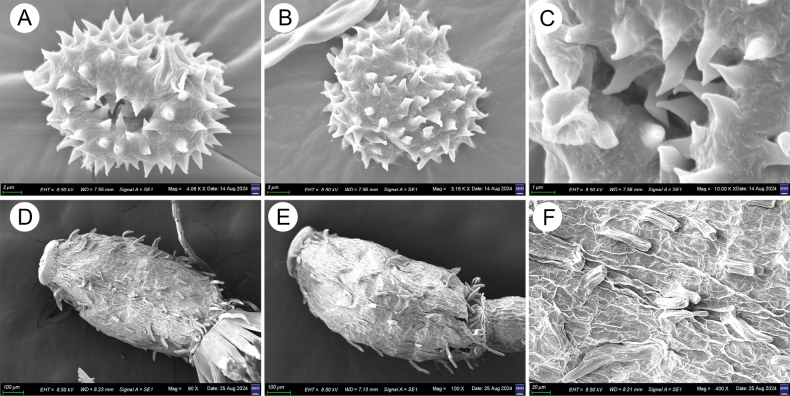
*Leontopodiumnyingchiense* X.Z.Lan, W.L.Zheng & W.Q.He **A–C** morphological structure of pollen under SEM **D–F** morphological structure of seeds under SEM.

China • Xizang (Tibet) Autonomous Region, Nyingchi city, Lulang town, alt. ca. 4440 m, 29°40.82'N, 94°46.71'E (DDM), 24 July 2015, *Xiao-Zhong Lan, Lian-Qiang Li 542621150724809LY* (holotype: XZE!; isotype: KUN! barcode 1628268) (Figs 1–5, Table 3).

**Table 3.** Morphological comparisons between *Leontopodiumnyingchiense* and *L.lingianum*.

Characters	*Leontopodiumnyingchiense*	*Leontopodiumlingianum*
Plant	monoecious or dioecious	dioecious
Involucre	involucral bracts in 3–5 series: the outer layer herbaceous, the middle and inner series membranous	involucral bracts multi-layered, densely imbricate, similar in shape to the leaves
Floret	apex of the female floret corolla brown, with fimbriate and lacerate margins; lobes of the male floret corolla with rough-edged margins	upper part of the corolla densely covered with white hairs
Achene	without a costa	with a costa

##### Diagnosis.

*Leontopodiumnyingchiense* is morphologically most similar to *L.lingianum* but can be distinguished by several features. The species includes monoecious and dioecious individuals, with some plants having both male and female flowers in the same individual. Its involucral bracts are in 3–5 series; the outermost series is herbaceous, while the middle and innermost series are membranous. The female floret corolla has brown, fimbriate apices and a fringed, ragged tip, while the male floret corolla has lobes with rough, granulate edges. Additionally, the achenes lack costae.

##### Description.

Perennial, pulvinate, caespitose herb, monoecious or dioecious, less than 10 cm tall. Leaves alternate or verticillate, imbricate, sessile, nearly sheathless at the base, elliptic, entire; apex herbaceous, both surfaces silky lanate; lower part membranous and glabrous, white with a reddish tint when young, turning brownish upon maturity, 3–12 mm × 1.6–2.5 mm. Capitula with uniform florets, either all male or all female, or bisexual florets, solitary at the apex of stems and branches, nearly sessile, immersed among leaves or slightly extending beyond them; ebracteate. Involucre hemispherical, 3–5 mm in diameter; phyllaries imbricate, in 3 series in male or monoecious capitula, in 5 series in female capitula; outermost bracts spatulate, entire, herbaceous, covered on both surfaces with lanate indumentum; middle and inner bracts 3–6 mm × 0.8–3 mm, lanceolate or linear, with fimbriate, lacerate margins, membranous, covered on the abaxial surface with lanate indumentum. Receptacle alveolate, with irregularly edged pits. Female florets fertile; corolla tubular, 2.5–3 mm long, 4-lobed at the apex, with brown fimbriate, lacerate margins. Male florets (or bisexual florets) do not produce viable seeds, with a non-functional ovary; corolla tubular, 1–3 mm long, 5-lobed at the apex, with short triangular lobes and rough-edged margins. Anthers linear, tailed at the base, without appendages at the apex; filaments free; stigma apex blunt. Achenes obovate-oblong, puberulous, ca. 1 mm long; sterile ovary ca. half the size of the achene, glabrous. Pappus in one layer, persistent, white, often serrulate, the upper part slightly thickened in male florets.

##### Phenology.

Flowering from July to early August, fruiting from late August to mid-September.

##### Etymology.

The epithet indicates the type locality, i.e. Nyingchi area, Xizang, China.

##### Vernacular name.

lín zhī huǒ róng cǎo (Chinese pronuciation); 林芝火绒草 (Chinese name).

##### Distribution and habitat.

*Leontopodiumnyingchiense* was discovered in Lulang Town, Nyingchi City, Xizang, China. This new species grows in alpine meadows at elevations of approximately 4400–4800 meters.

##### Conservation status.

The species appears to be narrowly distributed, currently known only from alpine meadows near Dongbazai Village in Lulang Town, Nyingchi City, with approximately 300–400 individuals observed (a total of less than 1,000 individuals). The habitat of the Nyingchi Edelweiss is susceptible to disturbance or degradation. Further field investigations are needed to assess the precise distribution of the species, and it is possible that other populations might be found in similar habitats such as the Sejila Mountains. Therefore, we provisionally classify this species as Data Deficient (DD) according to the International Union for Conservation of Nature (IUCN 2024) criteria.

##### Additional specimens examined

**(paratypes).** China • Xizang (Tibet) Autonomous Region, Nyingchi city, Lulang town, Dongbazai Village, alt. ca. 4650 m, 29°40.08'N, 94°47.08'E (DDM), 10 Aug. 2024, *Wen-qi He, Lian-Qiang Li & Guo-Zhu Chai 542621240810002LY* (KUN!, barcode 1628269, ♂) • alt. ca. 4601 m, 29°40.07'N, 94°47.07'E (DDM), 10 Aug. 2024, *Wen-qi He, Lian-Qiang Li & Guo-Zhu Chai 542621240810032LY* (XZE!, ♀).

### ﻿Molecular phylogeny

**Figure 6.**
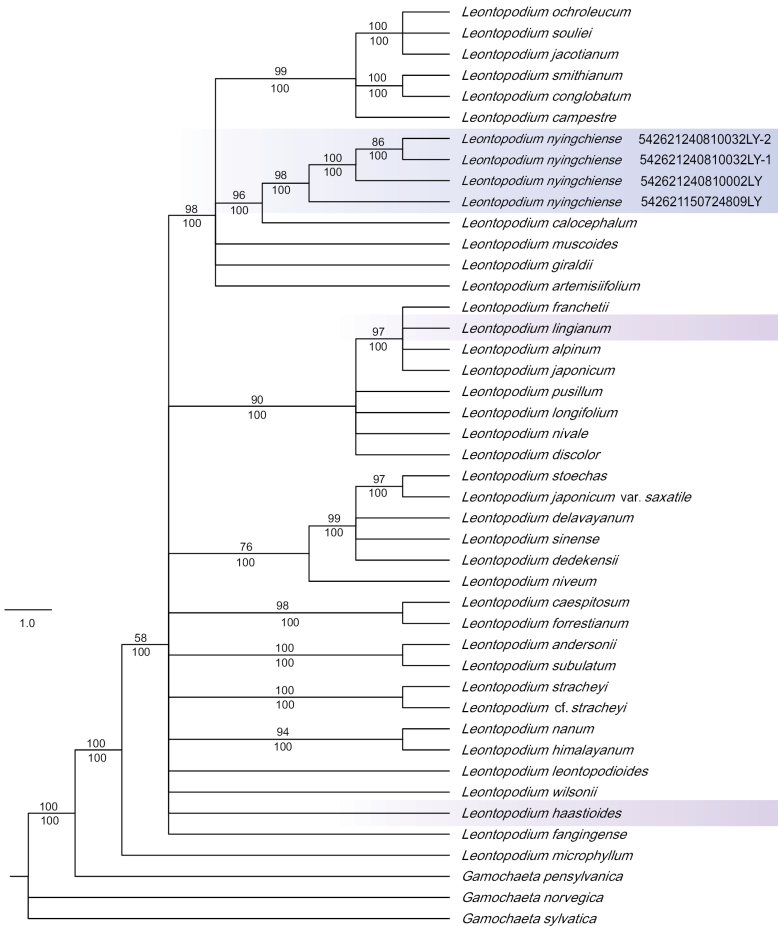
Bayesian consensus tree of *Leontopodiumnyingchiense* and related species based on ETS sequences. The tree is constructed using the ETS sequences. Numbers below the branches represent maximum likelihood bootstrap support (ML), and numbers above the branches represent Bayesian posterior probabilities. *Leontopodiumnyingchiense* is highlighted in blue.

Bayesian analysis (Bayes) and Maximum Likelihood (ML) analysis yielded similar phylogenetic trees (Fig. 6), with each branch annotated with the corresponding Bayesian posterior probabilities and ML bootstrap values. The phylogenetic results indicate that the four samples of the new taxon form a well-supported group (posterior probability = 98%, ML bootstrap = 100%). These samples are closely related to *Leontopodiumcalocephalum*, a relationship that is further supported by the phylogenetic tree constructed in this study. Previous studies by Blöch et al. (2010) and Xu et al. (2023) demonstrated that *Leontopodium* is a monophyletic group, a finding that is consistent with our results.

## ﻿Discussion

According to the phylogenetic analysis, *Leontopodiumnyingchiense* and *L.calocephalum* are very closely related, though they can be easily distinguished morphologically. The primary morphological distinction is that *L.nyingchiense* is a dwarf cushion plant and lacks involucral bracts. Morphologically, *L.nyingchiense* is most similar to *L.lingianum*, followed by *L.haastioides*. All three species are dwarf cushion plants and also lack involucral bracts, though *L.nyingchiense* differs from *L.haastioides* in that it does not produce rhizomes.

*Sinoleontopodium* was first described as a new genus by Chen (1985), but it was merged into the genus *Leontopodium* by Dickoré (Blöch et al. 2010), a classification accepted by POWO (Plants of the World Online https://powo.science.kew.org/). This classification was supported by our phylogenetic analysis using ITS and ETS sequences, which positioned this genus within *Leontopodium*. However, morphologically *L.lingianum*, *L.nyingchiense* and two species in the dense cushion group, *L.haastioides* and *L.aurantiacum*, are distinctly different from the other species in the genus due to the typical character of *Leontopodium*: prominent involucral bracts. This morphology suggests that *L.lingianum*, *L.nyingchiense* and the dense cushion group may warrant classification as a separate genus or subgenus. However, the phylogenetic results presented in this study indicate that, except for *L.aurantiacum* (for which molecular data is unavailable), the remaining three species are only distantly related. Therefore, with more molecular data, the classification of these species within the genus may need to be reassessed.

Field observations of *L.nyingchiense* revealed both monoecious and dioecious individuals. Typically, monoecious individuals have one male floret in the center of the female inflorescence, with the male floret being much smaller than the female florets. In dioecious individuals, male inflorescences are slightly larger than female ones, with the male florets being concealed among the leaves, while female inflorescences are located above the leaves. However, our dissection of samples in the laboratory revealed that in male plants, a small number of female florets occur within male inflorescences without any discernible pattern. These female florets are morphologically identical to those on female plants, possessing only a pistil with no stamens, and develop normally. In contrast, the male florets are functionally bisexual, with the pistil showing abnormal development, the ovary being sterile, and possessing five stamens. Therefore, in functional terms, this species lacks true male florets, possessing only bisexual and female florets. Under natural conditions, some fertile bisexual florets may occur, but the sample size in this study was insufficient, and further research is required.

## Supplementary Material

XML Treatment for Leontopodium nyingchiense

## ﻿Additional information

### Author ORCIDs

Wen-Qi He https://orcid.org/0009-0008-3041-5422

Fang-Yu Zhao https://orcid.org/0000-0001-6352-2955

Zhao-Fu Chu https://orcid.org/0000-0002-1069-3256

Guo-Zhu Chai https://orcid.org/0009-0000-9042-9668

Kai-Hui Zhao https://orcid.org/0000-0002-6665-2648

Fang-Yuan Zhang https://orcid.org/0000-0003-3840-9947

Zhi-Hua Liao https://orcid.org/0000-0002-1283-4649

Xiao-Zhong Lan https://orcid.org/0000-0002-8657-5790

### Data availability

All of the data that support the findings of this study are available in the main text.
